# In ovo feeding of methionine affects antioxidant status and growth-related gene expression of TETRA SL and Hungarian indigenous chicks

**DOI:** 10.1038/s41598-024-54891-3

**Published:** 2024-02-22

**Authors:** James K. Lugata, Sawadi F. Ndunguru, Gebrehaweria K. Reda, Gabriella Gulyás, Renáta Knop, János Oláh, Levente Czeglédi, Csaba Szabó

**Affiliations:** 1 Department of Animal Nutrition and Physiology, Faculty of Agriculture and Food Sciences and Environmental Management, University of Debrecen, Böszörményi Street 138, 4032 Debrecen, Hungary; 2 Department of Animal Husbandry, Faculty of Agriculture and Food Sciences and Environmental Management, University of Debrecen, Böszörményi Street 138, 4032 Debrecen, Hungary; 3 Doctoral School of Animal Science, Faculty of Agriculture and Food Sciences and Environmental Management, University of Debrecen, Böszörményi Street 138, 4032 Debrecen, Hungary; 4 Department of Evolutionary Zoology and Human Biology, University of Debrecen, Faculty of Science and Technology, Egyetem Street 1, 4032 Debrecen, Hungary; 5 Institutes for Agricultural Research and Educational Farm, University of Debrecen, Böszörményi Street 138, 4032 Debrecen, Hungary

**Keywords:** Developmental biology, Molecular biology, Physiology

## Abstract

Methionine (Met) plays a substantial role in poultry due to its involvement in several pathways, including enhancing antioxidant status and improving growth performance and health status. This study examined how in ovo feeding of Met affects hatching performance, antioxidant status, and hepatic gene expression related to growth and immunity in the TETRA-SL LL hybrid (TSL) commercial layer and Hungarian partridge colored hen (HPC) indigenous genotypes. The eggs were injected with saline, DL-Met, and L-Met on 17.5 days of embryonic development. The results showed that the in ovo feeding of DL-Met significantly increased the hatching weight and ferric reducing the ability of the plasma (FRAP) compared with L-Met. The in ovo feeding of either Met source enhanced the liver health and function and hepatic antioxidant status of the chicks. The genotype’s differences were significant; the TSL genotype had better hatching weight, an antioxidant defense system, and downregulated growth-related gene expression than the HPC genotype. In ovo feeding of either Met source enhanced the chicks' health status and antioxidant status, and DL-Met improved the hatching weight of the chicks more than L-Met. Genotype differences were significantly evident in the responses of growth performance, antioxidant status, blood biochemical parameters, and gene expression to Met sources.

## Introduction

It is widespread in the modern commercial chicken setting for newly hatched neonates to be denied access to feed and water for 24–48 h due to hatching time, hatchery treatments, and transportation variations^1^. This forces the chicks to rely only on the nutrients present in the yolk sac reserves during this period, which is insufficient to provide the energy needed for rigorous growth and metabolism, and these chicks are prone to oxidative stress due to the increased production of free radicals^2,3^. In addition, heat stress can be caused in chick embryos by high metabolic and environmental heat generation during the later hatching phase, which also contributes significantly to the generation of free radicals^4^. Antioxidants are an essential line of defense against free radicals. Freshly deposited eggs, on the other hand, contain relatively low antioxidant content. This means that the embryo is constantly subjected to the negative consequences of oxidative stress. A healthy antioxidative condition during incubation is essential for hatchling viability. The balance between pro-oxidants and antioxidants during embryonic development plays a role in the rearing time of chickens.

The TETRA-SL LL hybrid (TSL) is a brown egg layer hybrid that is suitable for both intensive industrial and extensive systems, either kept in a cage and alternative systems. They are known for efficient and persistent egg production with an extended life cycle, high livability, feed efficiency, and high hatchability due to genetic improvement. TSL produces eggs with excellent internal and external egg quality. The average egg weight is 64.0–65.5 g for 52-week-old TSL hens^5,6^. Hungarian Partridge-Colored hen (HPC) is among the indigenous chicken breeds that have been conserved in the gene reserves by the Hungarian government since 1973. They are known for their dual-purpose function and palatable meat. HPC has good adaptability, scavenging ability, and disease resistance capacity^5,7^. These differences are due to the variations in the alleles and genes and the interaction between their phenotype and the environment and nutrition. There is a scarcity of information on the effect of nutrient manipulation in the eggs of the TSL and HPC on the antioxidant status and gene expression related to growth and immunity.

The in ovo injection strategy overcomes these issues, bridges the gap between hatching, and provides a tool to overcome the imbalance between antioxidants and pro-oxidants^8^. Studies have shown that the amnion is an effective site for the in ovo injection method, and the embryo ingests the amniotic fluid before pipping. Pipping refers to the first hole the chick makes using the small horn-like structure on the tip of its beak (called egg tooth) as it starts to hatch. Therefore, this procedure is sometimes referred to as in ovo feeding^9^. In ovo feeding provides nutrients to chicks at a critical stage, facilitating embryo development and post-hatch growth performance^10^. The high demand for protein for growth and reduction of the negative effect of oxidative stress during late embryonic development are achieved by the in ovo feeding of specific nutrients^1^. In ovo feeding of methionine (Met) has been shown not only to alleviate oxidative stress^1,2,11^ but also to be used as a source of amino acids for protein synthesis and, hence, to decrease protein-based gluconeogenesis in hatchlings^12^.

Methionine (Met) is crucial for regulating chicken growth; however, the precise mechanism remains unclear. Methionine is a functional amino acid that influences several aspects of chicken production including weight gain, feed conversion ratio, and carcass quality^13^. Methionine affects feed consumption and gene expression in broiler chicken hypothalamus^14^. Methionine stimulates milk protein synthesis in domestic pigeons from agricultural plants via the Janus-activated kinase/signal transducers and activators of transcription (JAK2-STAT5) signaling pathway^15^. Broiler chickens that received methionine supplementation grew faster and expressed different intestinal nutrition transport genes^16^. These results suggest that methionine significantly regulates poultry growth, although additional research is needed to fully understand the underlying mechanisms. In addition, Met is regarded as an immunomodulatory agent and hence, plays a critical role in resisting microbial infection and diseases^11,13^. Met increases the expression of the Toll-like receptor (TLR), which plays a critical role in pathogen pattern recognition and hence participates in immune activation and protects chicks against pathogens^2,17^.

Studies have shown that the growth hormone (GH) and insulin-like growth factor 1 (IGF1) interact with methionine to control poultry growth. GH-boosting peptides enhance myofiber development in broiler chickens, increase serum IGF-1 and GH levels, and regulate the expression of myomiR and muscle-specific mRNA^18^. GH and amino acid supply influence IGF-I production and gene expression in ovine hepatocytes^19^. Scattered transcriptional enhancers govern the expression of *IGF-1* gene, while GH activates Stat5b-binding elements and stimulates the transcription of IGF-I gene^20^.

Despite the above-detailed research, studies on the role of Met in regulating growth by in ovo feeding in layers still need to be conducted. Therefore, the main objective of this study was to investigate the effects of Met in ovo feeding on plasma biochemical parameters and gene expression related to the growth and immunity of commercial and indigenous layer genotype chicks. We hypothesized that in ovo feeding of different Met sources would differentially influence hepatic insulin-like growth factor 1 (*IGF1*), insulin-like growth factor 1 receptor *(IGF1R)*, growth hormone receptor *(GHR),* and toll-like receptor 4 (*TLR4*) gene expression in one-day-old chicks. In addition, we also hypothesized that in ovo feeding improves the health status of chicks in both genotypes.

## Methods

### Ethics approval for animal experiments

The experimental protocol was approved by the Committee of Animal Welfare of the University of Debrecen, Hungary (6/2021/DEMA’B). We confirm that all experiments were performed per the EU directive “Legislation for the protection of animals used for scientific purposes.” We confirm that authors complied with the ARRIVE guidelines. The experiment was conducted at the Kismacs Experimental Station of Animal Husbandry of the Institute of Agricultural Research and Educational Farm, University of Debrecen (Debrecen, Hungary) in June 2021.

### Eggs and incubation setup

Fertile Hungarian Partridge-Colored hen (HPC) eggs were obtained from the Institute of Agricultural Research and Education Farm (University of Debrecen, Debrecen, Hungary). We collected 210 freshly laid eggs (not older than four days) from the HPC flock that were 52–58 weeks old. TETRA-SL (TSL) eggs were procured from a commercial breeder flock at 52 weeks of age (TETRA Ltd., Bábolna, Hungary). The TSL and HPC eggs were uniformly sized, and thus presented the same average weight class. However, we did not weigh the eggs individually during setup in the incubator. Based on the normal rule of thumb, a day-old chick should weigh two-thirds or 67% of its initial egg weight. We calculated the initial egg weight and obtained average weight of 64.12 ± 5.62 g and 53.06 ± 5.42 for TSL and HPC genotype respectively. The eggs and incubator were disinfected with formalin before the set/incubation. Five hundred and seventy eggs (360 TSL and 210 HPC) were marked according to genotype and transferred into an incubator (PLM 3600, PL Machine Kft., Budapest, Hungary) with automatic egg turning every two hours. Eggs were incubated under standard conditions (37.8 °C and 50% relative humidity) from 1 to 17.5 days of incubation. From 17.5 to 21 days of incubation, the relative humidity was raised to 65–70%. The eggs were candled on days 10th and 17.5th day (before in ovo injection) of incubation and non-fertile eggs and eggs with dead embryos were removed from the incubator.

### DL and L-Met in ovo injection

We prepared 10 mg Met/mL 0.75% normal saline solution from DL-Met (DL-methionine, No. M9500, purity ≥ 99%, Sigma‒Aldrich, Merck KGaA, Darmstadt, Germany) and L-Met (L-methionine, No. 64319, BioUltra, purity ≥ 99.5%, Sigma‒Aldrich, Merck KGaA, Darmstadt, Germany). On the 17.5th incubation day, we randomly divided the embryonated eggs into eight treatment groups with four groups per genotype. Each group consisted of 30 eggs for the TSL genotype and 20 eggs for the HPC genotype, depending on the availability of embryonated eggs per genotype. Each genotype consisted of four treatment groups as follows: the first group served as a positive control with non-injected eggs (Control), the second group was injected with 0.5 mL 0.75% saline solution (NaCl) only and served as the sham control (Saline), the third group was injected with 5 mg of DL-Met (0.5 mL of the prepared solution) (DL-Met), and the fourth group was injected with 5 mg of L-Met (0.5 mL of the prepared solution) (L-Met). The in ovo injection protocol was carried out according to Chen et al.^21^ and Tombarkiewicz et al.^22^. In brief, we disinfected the surface of every egg on the broad end with 70% ethanol soaked with a cotton ball. Then, we created a small hole using an egg drill (0.5 mm diameter) and injected 0.5 mL of 10 mg Met/mL solution into the amniotic sac using a 23 gauge needle^22^. The hole was immediately sealed with hot paraffin and the eggs were transferred to a hatcher.

### Sample collection

The weights of the chicks on the day of hatching and sampling day (1-day-old chicks) were recorded, and the chicks were euthanized by cervical dislocation for blood and tissue sampling. In addition, chick, liver, and heart weight were recorded. Blood samples were collected from the jugular vein into ethylenediaminetetraacetic acid (EDTA)-coated tubes on the sampling day. The blood samples were centrifuged at 3000 × g for 10 min at room temperature to separate the plasma, and the plasma samples were stored at − 80 °C until further analysis. Additionally, the tissues (liver and pectoral muscle) were extracted and snap-frozen in liquid nitrogen before being transported to the laboratory and stored at − 80 °C until further analysis.

### Liver enzymes and kidney status indicator (AST, ALT, and uric acid) assay

Plasma samples were analyzed for aspartate aminotransferase (AST), alanine aminotransferase (ALT), and uric acid using an auto analyzer (Lab-Analyze 10261, OrvosTechnika Ltd., Budapest, Hungary). These parameters were analyzed according to the kit manufacturer’s instructions (AST: GOT16K, ALT: GPT17K and uric acid: UAC12, OrvosTechnika Ltd., Budapest, Hungary). Briefly, measurement were performed at room temperature, and it was ensured that the samples and reagents were at room temperature before and during the measurements. Three technical replicates were performed depending on the parameters, according to the manufacturer’s instructions. The first measurement was the endpoint (uric acid measurement), wherein a 12 μL sample was pipetted into uric acid reagents. The respective mixed sample with the reagent was then incubated for 5 min. For AST and ALT, the measurement was kinetic, and distilled water was used as a blank. In this method, a 50 μL sample was pipetted into the AST and ALT respective reagents, gently mixed, and measured directly. The units used for the measured parameters were μmol/L for uric acid and U/L for AST and ALT, respectively.

### Ferric reducing ability of plasma (FRAP) assay

The ferric-reducing ability of plasma (FRAP) was determined according to the kit manufacturer’s protocol (Ferric Antioxidant Status Detection Kit, Catalog Number EIAFECL2; Thermo Fisher Scientific). Briefly, this method is based on the oxidized form of Fe ^III^ being reduced to Fe ^II^ under acidic conditions with the formation of a blue-colored ferrous–tripyridyltriazine complex. The intensity of the color change determines the antioxidant capacity. The assay buffer concentrate (10× acetate buffer with stabilizers and preservatives) was used to prepare the 1× assay buffer by diluting 7 mL of assay buffer 10× with 63 mL of deionized water. Plasma samples were diluted by adding 20 µL of plasma to 40 µL of 1× assay buffer to make a total volume of 60 µL. After dilution, the sample was mixed well by hand and centrifuged for approximately 1–2 min to check for bubbles. All the samples were used within 2 h of dilution. Standards were diluted as follows: 20 µL of 10 mM ferrous chloride standard was added to one tube containing 180 µL of 1× assay buffer and labeled with 1000 µM FeCl_2_. Then, 100 µL of 1× assay buffer was added to each of the six tubes and labeled with 500, 250, 125, 62.5, 31.25, and 0 µM FeCl_2._ After that, serial dilutions of the standards were made as follows: 100 µL of 1000 µM FeCl_2_ solution into a 500 µM FeCl_2_ tube, 100 µL from 500 µM FeCl_2_ solution into a 250 µM FeCl_2_ tube, 100 µL from 250 µM FeCl_2_ solution into a 62.5 µM FeCl_2_ tube, and 100 µL of a 62.5 µM FeCl_2_ tube into a 31.25 µM FeCl_2_ tube. The solution was thoroughly mixed between steps and used within two hours of dilution. The FRAP color solution was prepared by taking 12.5 mL of 1× assay buffer, 1.25 mL of FRAP reagent A, and 1.25 mL of FRAP reagent B to make 15 mL of the solution.

First, 75 µL of the FRAP color solution was added to each well. Then, 20 µL of standard or diluted sample was added to the appropriate wells. Mixed gently by hand and centrifuged for 1–2 min in the platefuge. The plate were incubated for 30 min at room temperature. The absorbance was read at 560 nm, a standard curve was generated using four-parameter curve fitting, and linear regression curves provided the best standard curve fit. However, the blank-corrected absorbance was used before plotting. The concentration of unknown samples was obtained from the standard curve and expressed as micromolar (µM) FeCl_2_ equivalents.

### Total glutathione content (GSH) determination

Liver and muscle samples were homogenized under liquid nitrogen in a cooled mortar and pestle, and 20 mg was transferred into a new tube and then placed in a mini cooler (− 20 °C)^23^. Five hundred microliters of 5% sulfosalicylic acid (SSA) were added and vortexed to mix the sample before incubation for 10 min on ice. The homogenate was centrifuged at 17,000 × g (VWR Micro-Star 17R) for 10 min at 4 °C to precipitate proteins. The supernatant was transferred into a new tube and frozen at − 80 °C until further analysis. The concentration of glutathione (GSH) in the tissues was determined using a glutathione colorimetric detection kit (EIAGSHC, Invitrogen, and Thermo Fisher Scientific Carlsbad, CA 92008 USA) as described in our previous study^24^.

### Total antioxidant capacity (TAC) determination

Liver and muscle tissues were homogenized by grinding them under liquid nitrogen in a cooled mortar and pestle. Approximately 100 mg of fine-ground tissue was weighed and suspended in 1000 µL of ice-cold phosphate-buffered saline (PBS) (1:9, wt./vol). The TAC was measured in tissue homogenates (centrifuged at 15,000 × g, 4 °C, 10 min) using a kit (MAK187, Sigma‒Aldrich) and a microplate reader (570 nm), as described in our previous study^24^. TAC concentrations were calculated as Trolox equivalents (mM/mg tissue) using a standard graph.

### Gene expression analysis

#### Total RNA isolation and cDNA synthesis

Liver samples were homogenized using an Ultraturax homogenizer (D1000 handheld homogenizer; Benchmark Scientific, Inc., Sayreville, NJ, USA). The muscle tissues were homogenized by grinding them in liquid nitrogen in a cooled mortar and pestle. Total RNA was isolated using TRIzol reagent according to the Direct-zol RNA Miniprep (R2052; Zymo Research Orange, CA, USA) kit protocol. The quantity and purity of RNA were determined using a microplate reader (Synergy HT Multi-Mode Microplate Reader-SN 1712214, BioTek Instruments, Inc., Winooski, USA) as previously described^24^. The quality and integrity of RNA were checked using a Qubit RNA IQ assay kit (# Q33222, Thermo Fisher Scientific) measured using a Qubit 4 fluorometer (Invitrogen, Thermo Fisher Scientific). The RNA IQ numbers ranged from 8.7 to 10. cDNA synthesis was performed using the LunaScript RT SuperMix Kit (New England Biolabs Inc. E3010L) with 200 ng total RNA and a PCR^max^ Alpha Thermal Cycler (Cole-Parmer Ltd. UK). The cDNA was stored at – 80 °C until the RT-PCR assay.

#### Real-time PCR

The cDNA samples were amplified according to the manufacturer’s instructions using 5 × HOT FIREPol EvaGreen qPCR Master Mix Plus (Solid BioDyne, Tartu, Estonia). In brief, PCR with a total volume of 10 µL consisting of 2 ng cDNA template, 5 × HOT FIREPol EvaGreen qPCR mix plus 200 nM of each primer, and distilled water was used. An AriaMx Real-Time PCR system was used to perform real-time polymerase chain reaction (PCR) (Agilent Technologies Applied Biosystems, Carlsbad, CA, USA). The samples were run in duplicate in a 96-well plate, and no template control for each gene was used. The PCR procedure included a pre-run at 95 °C for 12 min, followed by 40 cycles of denaturation at 95 °C for 15 s, an annealing at 60 °C for 20 s, and elongation at 72 °C for 20 s. 18S ribosomal RNA (18S rRNA) was selected as the reference gene among the three reference genes tested (β-actin *-ACTB* and glyceraldehyde-3-phosphate dehydrogenase *-GAPDH*) and its stability was tested using the following algorithms (NormFinder, delta Ct, and Best Keeper). The target gene mRNA expression was normalized with the selected reference gene, and the relative mRNA expression was calculated using the 2^−ΔΔCt^ models^25^. Melting curves revealed no nonspecific product or primer dimers, suggesting the accuracy of mRNA transcript identification by displaying *IGF1, IGF1R, GHR,* and *TLR4* specific primers suitable for RT‒PCR (Table 1).

**Table 1:** Primer details of the target and reference genes.

Genes^a^	Primer sequence (5′–> 3′)	GenBank accession No.	Product length (bp)
*IGF1*_F	CAC TAT GCG GTG CTG AGC TGG TT	XM_015867574.2	118
*IGF1*_R	ATC CCC TTG TGG TGT AAG CGT CT		
*IGF1R*_F	TAC AAC TAC CGC TGC TGG ACC AC	XM_015873184.2	107
*IGF1R*_R	AGG CAC TCA GGA TGG CAA CAC		
*GHR*_F	GGC ACT GGT CTG TGT GAA TGA CT	XM_032441512.1	89
*GHR*_R	CCA GCT CAG GTG ATC TGC ACT T		
*TLR4*_F	ACCCGAACTGCAGTTTCTGGAT	NM_001030693.1	120
*TLR4*_R	AGGTGCTGGAGTGAATTGGC		
*18S rRNA*_F	CTC TTT CTC GAT TCC GTG GGT	AF173612.1	96
*18S rRNA*_R	CAT GCC AGA GTC TCG TTC GT		

^a^*IGF1*, insulin-like growth factor 1; *IGF1R*, insulin-like growth factor 1 receptor; *GHR*, growth hormone receptor; *TLR4*, toll-like receptor 4; 18S rRNA, 18S ribosomal RNA.

### Statistical analysis

All statistical analyses were performed using R version 4.2.2^26^. An individual bird (chick) was considered an experimental unit for all parameters. Data were analyzed using two-factor analysis of variance (ANOVA), and a general linear model was appropriate for evaluating the fixed effects (genotype and Met sources) and their interactions. When the interaction effect was significant, the treatment effect was analyzed separately for each genotypes. Tukey’s post hoc test was performed to compare the mean differences between the treatments. Linear discriminant analysis was performed between hepatic gene expression and hatching body weight, liver weight, and heart weight. The significance level for differences was set at *P* < 0.05.

## Results

### Effect of in ovo feeding of methionine sources on hatching weight, liver weight and heart weight

The L-Met group showed a significant decrease in both chick hatching weight and absolute heart weight compared with the DL-Met and control groups (Table 2, *P* < 0.05). The genotype significantly affected the hatching weight (*P* < *0.001*), heart weight (*P* = *0.029*), and relative liver weight *(P* = *0.032).* The data shows that the TSL genotype exhibited higher hatching and heart weight, while simultaneously demonstrating lower relative liver weight in comparison to the HPC genotype. There was no significant interaction between treatment and genotype, except for the absolute liver weight (Table 2, *P* = *0.029*)*.* In the TSL genotype, the L-Met group showed significantly reduced hatching weight, while no significant effect of the treatment was observed in the HPC genotype.

**Table 2:** Effect of in ovo feeding of methionine on hatching body weight, liver and heart absolute and relative weight of TSL and HPC chicks.

Parameter		HBW (g)	ALW (g)	AHW (g)	RLW (%)	RHW (%)
Genotype n = 32						
	TSL	41.24^a^	1.17	0.37^a^	2.84^b^	0.90
	HPC	35.75^b^	1.15	0.33^b^	2.97^a^	0.92
Treatment, n = 8						
	Control	39.66^a^	1.17	0.36^a^	2.99	0.92
	Saline	38.23^ab^	1.17	0.34^ab^	3.05	0.90
	DL-Met	39.45^a^	1.21	0.37^a^	3.08	0.95
	L-Met	36.46^b^	1.10	0.32b	3.02	0.88
*p Value*	Genotype	** < 0.001**	0.636	**0.029**	**0.032**	0.164
	Treatment	**0.035**	0.503	**0.038**	0.901	0.565
	Interaction	0.219	**0.029**	0.349	0.063	0.374
RMSE		3.291	0.229	0.015	0.578	0.132
Model		< **0.001**	0.111	**0.026**	0.091	0.42
Treatment effect by genotype						
	n = 8					
TSL	Control	43.59^a^	1.20	0.38	2.78	0.88
	Saline	40.73^a^	1.29	0.37	3.15	0.92
	DL-Met	41.37^a^	1.12	0.37	2.71	0.89
	L-Met	38.34^b^	1.08	0.34	2.81	0.88
*P-value*		**0.006**	0.221	0.280	0.335	0.876
RMSE		2.70	0.21	0.05	0.51	0.11
HPC	Control	35.69	1.11	0.35	3.16	0.98
	Saline	35.48	1.00	0.31	2.84	0.89
	DL-Met	37.71	1.33	0.37	3.51	0.99
	L-Met	34.81	1.12	0.31	3.22	0.88
*P-value*		0.467	0.091	0.073	0.232	0.306
RMSE		3.79	0.25	0.06	0.64	0.15

Significant *p*-values are in [bold].

Means with a similar letter(s) are not significantly different within the column and effect (P > 0.05).

RMSE, root square of the mean square of error; TSL, TETRA SL genotype; HPC, Hungarian partridge colored hen genotype; HBW, hatching body weight; ALW, absolute liver weight; AHW, absolute heart weight; RLW, relative liver weight; RHW, relative heart weight.

### Effect of treatments on the plasma biochemical parameters

Treatment with in ovo feeding of Met sources significantly influenced plasma uric acid, AST, ASL/ALT ratio, and FRAP (*P* < *0.05*). Additionally treatment tended to influence ALT levels (*p* = *0.056*) (Table 3)*.* In ovo feeding with L-Met significantly lowered the levels of uric acid and FRAP (*P* < *0.05*) in the plasma compared with the control group. In addition, the L-Met group had significantly lower FRAP content than the DL-Met group (*P* < *0.05*). The saline group had a significantly higher AST level than the DL-Met, and a higher ASL/ALT ratio than the other groups (*p* < *0.05,* Table 3)*.*

**Table 3:** Effect of in ovo feeding of methionine on the 17.5th day of incubation on blood biochemical parameters of TSL and HPC one-day chicks.

Plasma parameter		AST (U/L)	ALT (U/L)	AST: ALT	Uric acid (µmol/L)	FRAP (µM FeCl_2_ equivalent)
Pooled effects						
Genotype n = 32						
	TSL	93.63	57.00	1.64^b^	301.73^a^	78.96^a^
	HPC	98.08	59.50	2.02^a^	267.67^b^	70.27^b^
Treatment n = 8						
	Control	93.20^b^	63.63	1.42^b^	323.1^a^	81.36^a^
	Saline	109.13^a^	49.90	2.81^a^	283.99^ab^	73.01^a^
	DL-Met	88.11^b^	63.59	1.43^b^	284.22^ab^	84.80^a^
	L-Met	93.12^b^	55.89	1.66^b^	247.49^b^	59.30^b^
p-Values	Genotype	0.2750	0.6820	**0.0340**	**0.0391**	**0.0171**
	Treatment	**0.0017**	0.0563	**0.0001**	**0.0110**	**0.0002**
	Interaction	**0.0007**	0.0772	0.2331	**0.0001**	**0.0021**
RMSE		15.17	16.35	0.82	59.74	14.88
Treatment effect by genotype n = 8						
TSL	Control	102.22	57.23	1.71	329.44^ab^	90.12^a^
	Saline	94.09	57.56	1.56	365.36^a^	87.39^a^
	DL-Met	87.54	63.47	1.42	276.59^b^	78.21^a^
	L-Met	91.19	49.73	1.87	235.54^c^	60.12^b^
	P-value	0.3200	0.3584	0.2361	**0.0075**	**0.0069**
	RMSE	15.97	15.024	0.4422	61.93	16.89
HPC	Control	84.18^b^	70.02^a^	1.12^b^	316.78^a^	72.60^b^
	Saline	124.16^a^	42.23^b^	4.06^a^	202.61^b^	58.62^b^
	DL-Met	88.67^b^	63.71^ab^	1.44^b^	291.85^a^	91.38^a^
	L-Met	95.06^b^	62.03^ab^	1.45^b^	259.43^ab^	58.48^b^
	P-value	**0.0001**	**0.0267**	**0.0001**	**0.0035**	**0.0001**
	RMSE	14.26	17.58	1.0761	57.81	12.34

Significant *p*-values are in [bold].

Means with similar superscripts are not significantly different within the column and effect (P > 0.05).

RMSE, root square of the mean square of error; TSL, TETRA SL genotype; HPC, Hungarian partridge colored hen genotype; AST, aspartate aminotransferase; ALT, alanine aminotransferase—AST; ALT, aspartate aminotransferase alanine aminotransferase ratio; FRAP, ferric reducing ability of the plasma.

The saline group exhibited significantly lower (*P* < *0.05*, Table 3) plasma uric acid levels than the control and DL-Met groups for the HPC genotype. In contrast, the L-Met group had significantly decreased plasma uric acid levels in the TSL genotype (*P* < *0.05*, Table 3*).* The saline in ovo injection significantly increased the AST level and AST: ALT ratio compared to all other treatment groups (*P* < *0.05,* Table 3), but significantly reduced the ALT level when compared to the control group in the HPC genotype (*P* < *0.05,* Table 3). However, in the TSL genotype, no significant effect of treatment was observed on either AST or ALT levels or the AST: ALT ratio (*P* > *0.05*). The DL-Met in ovo injection significantly increased the FRAP content in HPC chicks compared to the other groups (*P* < *0.05*). However, in the TSL genotype, L-Met in ovo injection significantly reduced the FRAP content more than the other experimental groups (*P* < *0.05,* Table 3).

The genotypes did not significantly influence plasma parameters, except for the AST: ALT ratio, uric acid, and FRAP content (*P* < *0.05,* Table 3). The TSL genotype had significantly higher uric acid and FRAP content than the HPC genotype. In contrast, the HPC genotype had a higher AST: ALT ratio in the saline group than that in the TSL genotype (*P* < *0.05)*. Significant differences in AST and FRAP levels between genotypes were observed only in the control and saline groups (*P* < *0.05).*

### Glutathione and total antioxidant concentration in the liver and muscles

**Figure 1:**
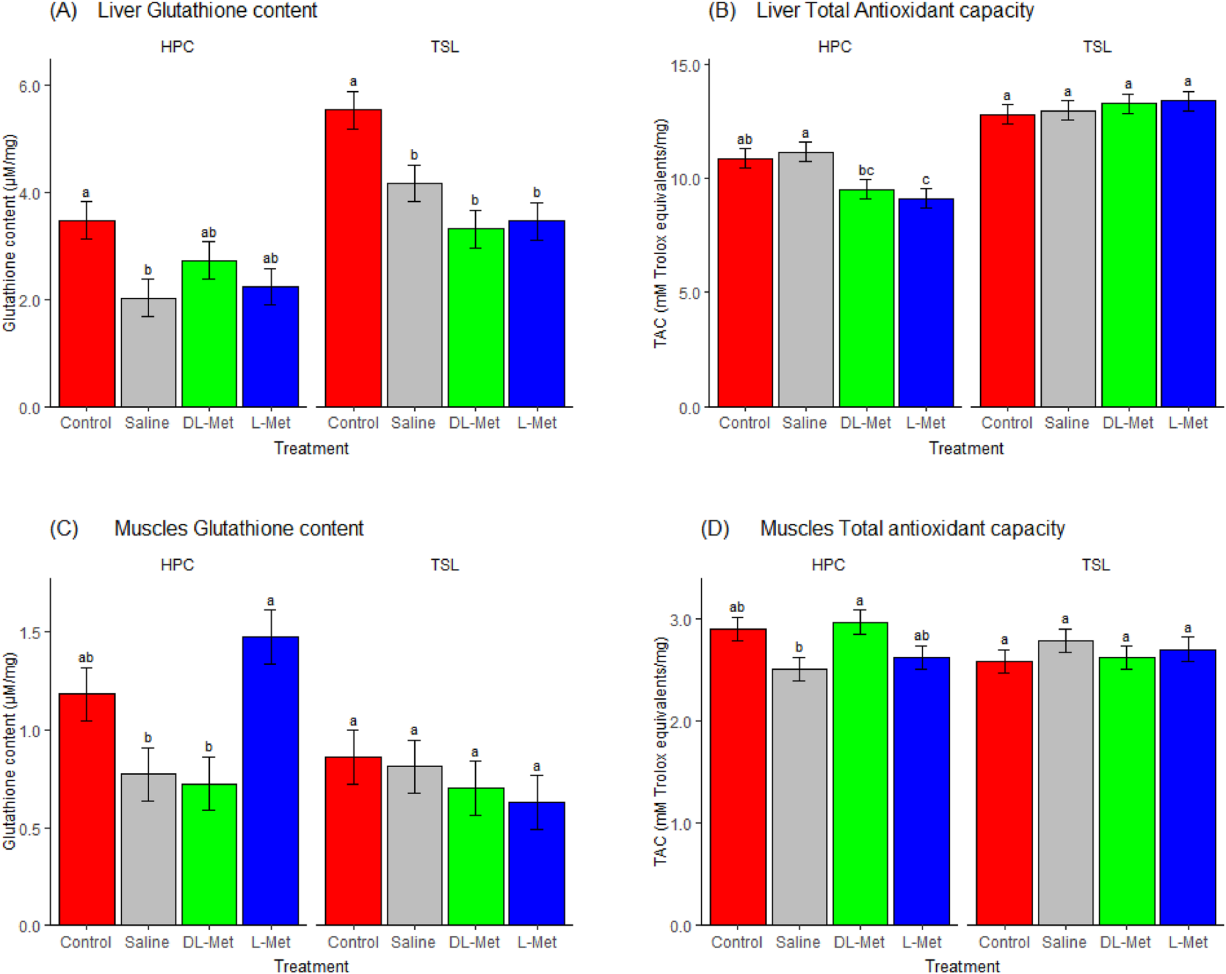
Influence of in ovo injection of Met on the liver glutathione and total antioxidant capacity of TSL and HPC chicks at one day old. TSL, TETRA SL genotype; HPC, Hungarian partridge colored hen genotype. (**A**) Liver glutathione content, (**B**) liver total antioxidant capacity, (**C**) muscle glutathione content, (**D**) muscle total antioxidant capacity. Means with similar superscripts are not significantly different (*P* > *0.05*) (Means ± SEMs, n = 8).

In ovo feeding and the genotype significantly affected the glutathione content of the chicks’ livers one day post-hatching (*P* < *0.05*). No interaction effect was observed for liver glutathione content (*P* > *0.05*). Tukey’s multiple comparison tests revealed that the genotype responded differently to in ovo feeding of methionine, with DL-Met and L-Met decreasing the glutathione content in TSL genotype chicks when compared to the control group (*P* < *0.05*, Fig. 1A). L-Met, DL-Met and saline resulted in a significantly reduced hepatic GSH content in HPC chicks compared to the control (*P* < *0.05*). Unlike the glutathione content, the hepatic TAC level was not affected by the in ovo feeding of Met to the TSL chick liver. The genotype and the interaction of genotype and treatment effects on TAC concentration were significantly different (*P* < *0.05*). The treatment had no significant effect on TAC levels in the livers of the TSL chicks. Nevertheless, the in ovo feeding of L-Met significantly reduced the TAC level in the liver of HPC genotype chicks compared to the control group (*P* < *0.05*). The saline-injected group had a significantly higher TAC content in the liver of HPC than the DL-Met (*P* = *0.037*) and L-Met (*P* = *0.006*) groups (Fig. 1B).

**Figure 2:**
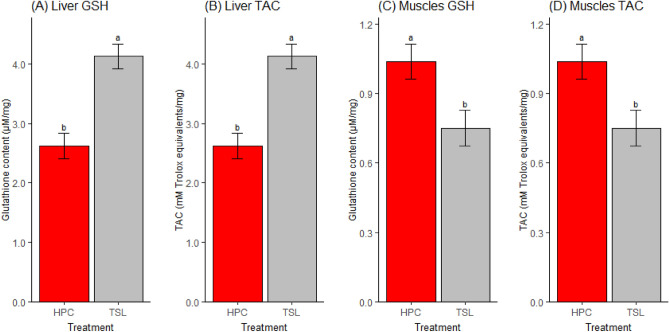
Genotype significantly influences the glutathione and total antioxidant capacity concentration in the liver and muscles. GSH, glutathione; TAC, total antioxidant capacity; TSL, TETRA SL genotype; HPC, Hungarian partridge colored hen genotype. (**A**) Liver glutathione content, (**B**) liver total antioxidant capacity, (**C**) muscle glutathione content, (**D**) muscle total antioxidant capacity. Means with similar superscript letters are not significantly different (*P* > *0.05*) (Means ± SEMs, n = 32).

The muscle glutathione content was significantly affected by treatment, genotype, and interaction (*P* < *0.05).* The in ovo feeding of L-Met significantly increased the glutathione content more than the DL-Met and the saline groups in the muscles of HPC chicks (*P* < *0.05*). Nevertheless, no significant effects were observed in the TSL chicks (Fig. 1C). Muscle TAC concentration was only affected by the interaction between genotype and treatment (*P* < *0.05*). *The* DL-Met group had a significantly higher TAC concentration than the saline group (*P* = *0.035*) in the muscles of the HPC chicks. No significant effects were noted in TSL chicks (Fig. 1D). Regarding the genotype, HPC chicks had significantly higher glutathione and TAC levels in the muscles than the TSL chicks. In the liver, the opposite pattern was observed; TSL chicks had significantly higher glutathione and TAC content than HPC chicks (*P* < *0.05,* Fig. 2)*.*

### Effect of in ovo feeding of different methionine sources on gene expression

**Figure 3:**
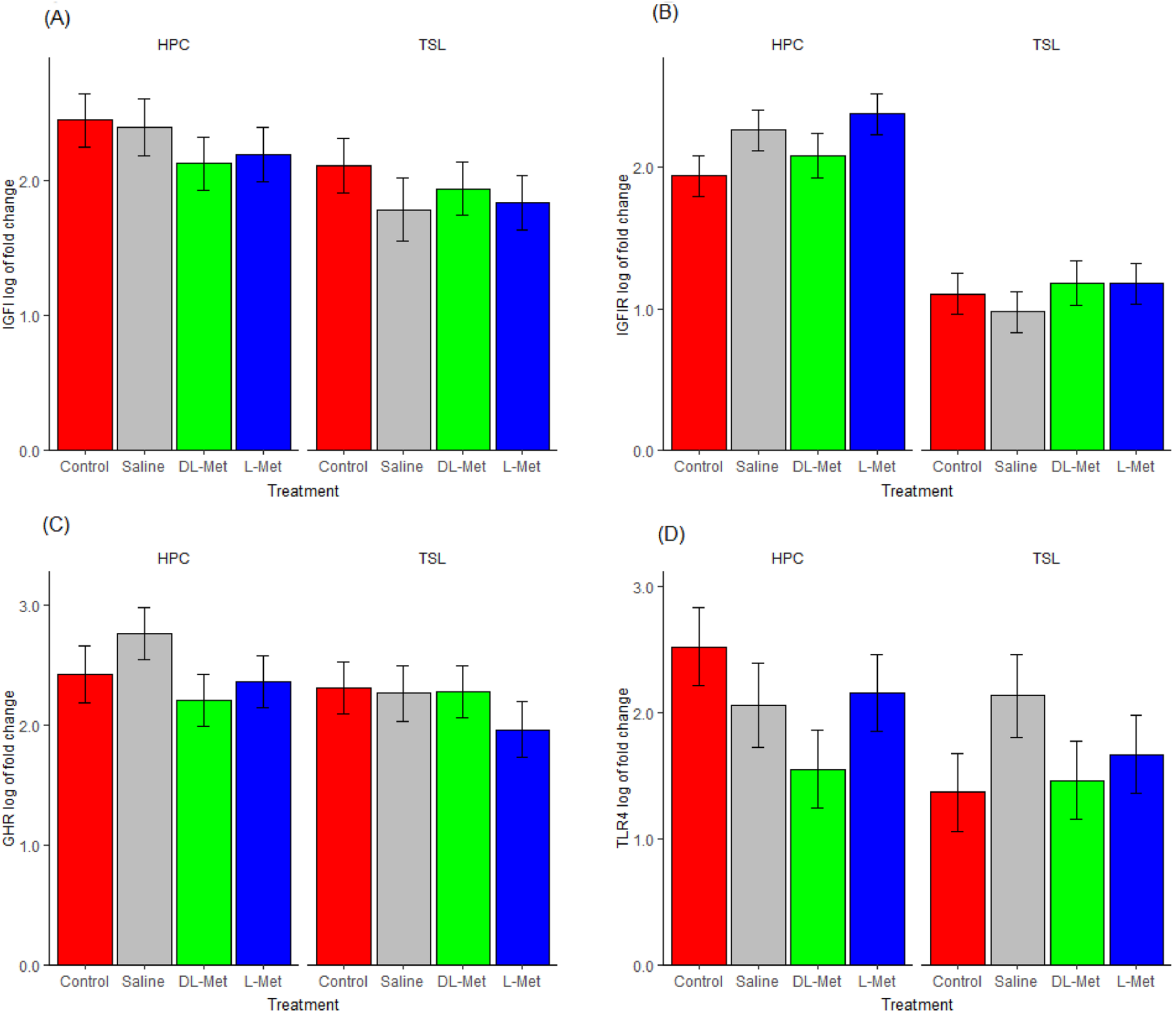
Effect of in ovo feeding of methionine on the liver gene expression of TSL and HPC chicks. (**A**) Insulin-like growth factor 1 gene expression. (**B**) Insulin-like growth factor 1 receptor gene expression. (**C**) Growth hormone receptor gene expression. (**D**) *TLR4* gene expression. TSL, TETRA SL genotype; HPC, Hungarian partridge colored hen genotype; *IGF1*, insulin-like growth factor 1; *IGF1R*, insulin-like growth factor 1 receptor; *GHR*, growth hormone receptor; *TLR4*, toll-like receptor 4 (Means ± SEMs, n = 8).

There were no significant influences on the expression of the studied genes in the liver of one-day-old chicks by the in ovo feeding of methionine (Fig. 3A–D). However, chicks responded differently to the in ovo feeding of Met sources within the genotypes. In the HPC genotype, in ovo feeding of L-Met tended to upregulate the expression of *IGF1R* (P = 0.16, Fig. 3B), whereas DL-Met tended to downregulate the expression of *TLR4* (P = 0.12, Fig. 3D) compared to the control. No significant effect or tendency in the expression of the genes (*IGF1, IGF1R, GHR*, and *TLR4*) was noted for TSL genotype chicks (Fig. 3A–D).

### The genotype effect on gene expression

**Figure 4:**
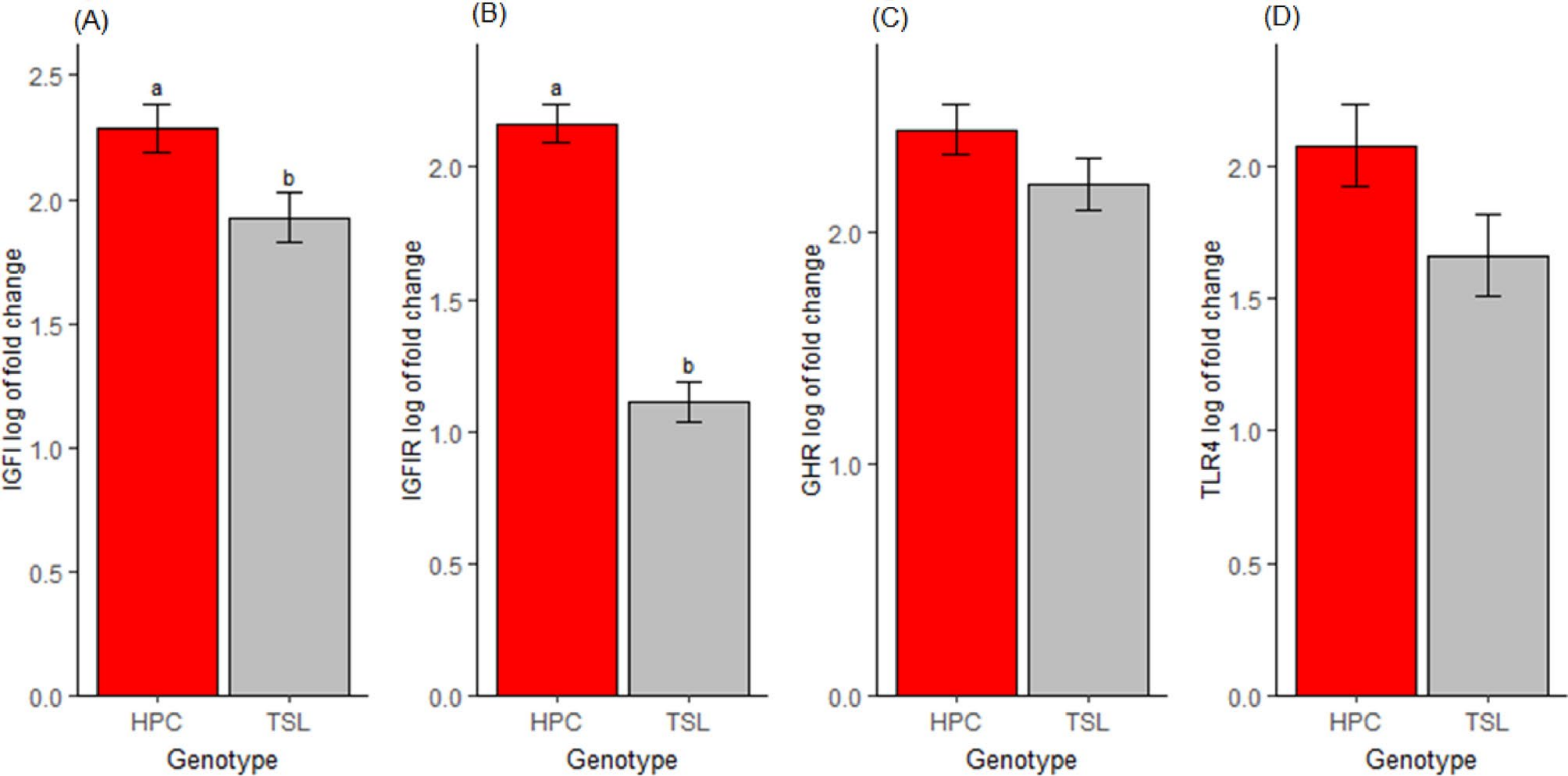
Hepatic gene expression between the two genotypes. (**A**) Insulin-like growth factor 1 gene expression. (**B**) Insulin-like growth factor 1 receptor gene expression. (**C**) Growth hormone receptor gene expression. (**D**) *TLR4* gene expression. Means with different superscript letters are significantly different (P > 0.05). (Means ± SEMs, n = 32).

The genotype significantly influenced the expression of the *IGF1* and *IGF1R* genes (*P* < *0.05*) and had no significant (only tended to) influence on *TLR4* (*P *= 0.058) and *GHR* gene expression (P = 0.15) (Fig. 4). No interaction effect of genotype and in ovo feeding on gene expression. Regarding the effect of genotype in the respective treatment groups, no significant differences were observed, except for the *IGF1R* gene (*P *< 0.001). *TLR4* gene expression differed significantly between genotypes (*P* = 0.011) in the control group.

### Liver discriminant analysis between gene expression and hatching body weight, liver weight and heart weight

**Figure 5:**
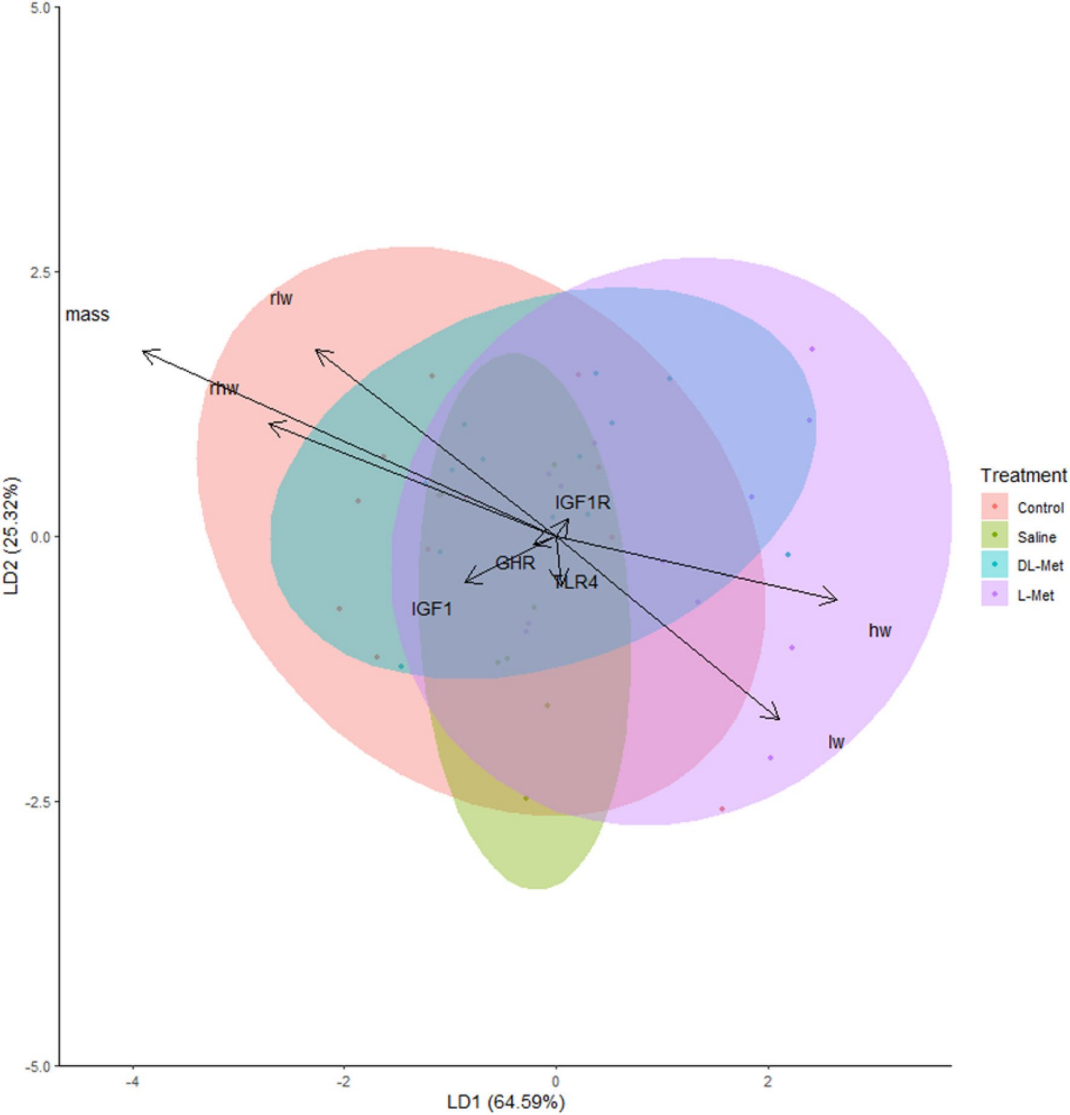
Linear discriminant analysis of hatching performance and gene expression parameters as the responses to in ovo feeding of Met during incubation. The ellipses in the figure represent the linear discriminant analysis’s 95% confidence interval for predicting sample classification. The parameters measured included mass-hatching body weight, heart weight (hw), liver weight (lw), relative heart weight (rhw), relative liver weight (rlw), insulin-like growth factor 1 receptor (IGF1R), insulin-like growth factor 1 (IGF1), growth hormone receptor (GHR), and toll-like receptor 4 (TLR4).

Using gene expression and hatching performance, linear discriminant analysis (LDA) was ineffective in discriminating among the four treatment groups (Fig. 5). LD1 (the first and the second linear discriminant axes represent 64.59% and LD2 25.32% of the total dispersion in gene expression and performance parameters among the treatment. The first discriminant vector (LD1) indicated that the liver (lw) and heart (hw) weights had the highest positive coefficients. The hatching body relative to liver and heart weights had the highest negative coefficients (− 3.91, − 2.11, and − 2.66, respectively) (Fig. 5). This means that the treatments were best discriminated by the hatching performance parameters. All gene expression variables had small discrimination coefficients ranging from − 0.21 to 0.11, indicating their relatively poor contribution/influences in discriminating between the control, saline, DL-Met, and L-Met treatment groups. The second and third discriminant functions contributed to 35% of the model variation, and the second LDA (LD2) had large coefficients of discrimination for body weight (1.75), liver weight (− 1.72), relative liver weight (1.76), and relative heart weight (1.06). The gene expression variables, such as LD1 and LD2, showed the least contribution in separating the treatments, with coefficients of discrimination of − 0.4 to 0.15.

## Discussion

Hatching body weight and heart weight were significantly improved by the in ovo feeding of DL-Met in this study compared to that of L-Met, but not significantly different from the control. These findings corroborate other studies that have shown that in ovo injection of Met increases the hatching weight of chicks^2,27^. The differences in hatching weight observed in this study may be attributed to the Met sources and genotypes studied. As mentioned, these studies used DL-Met in Rhode Island Red Breeders eggs^27^ and methionine plus cysteine in broilers^2^. The present study demonstrated that Met sources significantly affect the hatching weight and relative weight of important organs such as the heart, with L-Met in ovo feeding having a more negative effect than DL-Met. This might be due to the differences in physiological properties between the two sources; hence, their utilization by the growing embryo might differ^28^. This result indicates that L-Met might be less beneficial to layer-growing embryos and should be applied at a relatively lower dose than DL-Met.

Chicken-developing embryos are prone to oxidative stress, which negatively affects their development and post-hatching performance. The current study was designed to evaluate the effect of in ovo application of Met sources on improving chicken embryo antioxidants, health status, and growth. Chicken embryos have been found to have different antioxidant defense mechanisms to combat the effects of oxidative stress during the hatching period^29^. Uric acid is an important nitrogen metabolic end product in birds^30^. Additionally, it plays an essential role in plasma total antioxidant capacity; hence, it is used as an indicator of renal function/status^31^. In our experiment, in ovo injection of L-Met in the eggs significantly reduced the uric acid content and FRAP levels in the circulating blood of one-day chicks compared to the control. This result agrees with the findings of Wang et al.^33^, who found a linear decrease in uric acid content with the supplementation of either DL or L-Met, which reflected the better utilization of dietary nitrogen in broilers due to Met addition^30^. The high uric acid concentration in non-injected chicks could result from increased liver metabolic processes owing to imbalanced amino acids^30^.

The ferric-reducing ability of the plasma (FRAP) involves antioxidants that provide electrons to reduce ferric ions to their ferrous form. The higher the FRAP value, the stronger the antioxidant capability^34^, reflecting the plasma's water-soluble antioxidants^35^. Plasma FRAP comprises 60% uric acid, 15% ascorbic acid, 10% protein-SH groups, and 5% tocopherols^35,36^. In our trial, the in ovo injection of L-Met reduced the antioxidant power of chicks compared to the control and DL-Met-injected chicks. This corroborates the findings of^37^, who found no significant effect of increasing the L-methionine level from 100% of the National Research Council (NRC) to 150% of the recommendations. In contrast, significant effects were observed on FRAP between other sources (MHA and DL-Met) of methionine. The reduction of FRAP by in ovo injection of L-Met compared to DL-Met might be because L-Met was better utilized by enterocyte cells and used as a more efficient substrate for protein metabolism than DL-Met^38^. However, this is supported by the plasma uric acid results, since more than 50% of the plasma antioxidant power is due to uric acid. Additionally, L-Met is readily available for utilization by cells (protein synthesis intrakinases and oxidative stress) compared to DL-Met, in which the D-isomer must be converted to the L-isomer in the liver or kidney to be used by the cell^39^ because the critical enzyme is available in the liver or kidney. Therefore, D-Met is not readily available for use in the gastrointestinal cells. Interestingly, some studies have also indicated that the expression of this key enzyme is deficient in young animals. Moreover, in another experiment, the Met source, as well as the interaction of the source and the dose, has been indicated on FRAP (DL-Met vs MHA) with DL-Met elevating the plasma FRAP^40^.

Glutathione (GSH) is a potential antioxidant that plays a significant role in removing free radicals such as peroxides and hydroxyl radicals and plays a part in maintaining thiols from membrane proteins, in addition to acting as a substrate for GPx and glutathione reductase^41,42^. The hypothesis here was that in ovo injection with L-Met would positively enhance the redox status (GSH content) of chicks compared to DL-Met and the control, since L-Met could be immediately converted to Cys and then to GSH in the cytoplasm of hepatocytes. However, the results of this experiment do not concur with our hypothesis and do not corroborate other studies^2,43^. It has been established that the first Met-pass metabolism in the gut of a broiler affects its redox and growth performance^39^. In this experiment, the first-pass metabolism of Met by the gut of TSL and HPC chicks did not affect or improve their redox status by increasing total GSH content, as expected. A reduction in hepatic glutathione content was observed, and we assumed that there was a balance between the antioxidant scavenging system and reactive oxygen species (ROS) production, thereby facilitating chick embryo development. The imbalance between antioxidants and ROS production could arrest chick embryo development^44^. In line with our findings, Wang et al.^32^ reported that Met sources and levels influence liver GSH content in the diet. They further demonstrated that the Met source was correlated with reduced GSH content and had a protective role against ROS. In our experiment, both Met sources reduced GSH content in both genotypes, except L-Met, in the muscles of HPC chicks. Similar to the GSH content, the total antioxidant capacity of the liver and muscles was affected by the in ovo feeding of Met. L-Met significantly reduced TAC activity in the liver and tended to reduce muscle TAC in HPC chicks, whereas no significant effect was observed in the TSL. This result highlights the difference in response to the treatment between two genotypes.

Furthermore, in ovo feeding of DL-Met improved liver status, as it had the lowest mean AST (87.02 U/L) and AST: ALT ratio. In comparison, an increase in AST liver enzyme levels above 230 U/L indicates hepatic damage in birds^45^. The AST:ALT ratio and AST and ALT levels indicate liver damage in mammals and birds^45^. AST is considered a sensitive avian indicator of hepatic damage and muscle injury as its level positively correlates with tissue damage^46,47^. At the same time, ALT is a nonspecific cell damage indicator^47^. In our experiment, in ovo injection enhanced the hepatic status compared to the control group. This indicated a healthy functional liver, which was affected by the in ovo feeding of Met sources.

Another aim of this study was to evaluate the effect of methionine sources in ovo feeding on hepatic growth-related genes of the two chicken genotypes. This study demonstrated the interference of Met with gene expression in growth metabolic pathways. IGF-1 plays a significant role in chicken growth, with the lowest plasma IGF-1 levels decreasing growth rate^48^. GH plays a critical role in regulating the plasma concentration of IGF-1, with its essential control point being at the level of the liver and GH receptor/signal transduction^48^. Met sources have been shown to influence the expression of *IGF1* and *GHR* in broilers when supplemented in their diets at different concentrations^49^. Their trial revealed that broilers fed DL-Met at the highest level had the highest expression of *IGF1* and *GHR* genes and the best growth performance^49^. However, in our experiment, no significant effect was observed on the hepatic *IGF-1, IGF1R*, and *GHR* gene expression of either source of Met in ovo feeding on the eggs of either genotype. This result does not corroborate recent findings, which indicated that in ovo injection of Met-Cys increased the expression of *IGF1* in broiler chicks^43^. This difference from the above studies might be due to strain/genotype differences in response to Met treatment. Our current study explored two-layer genotypes, commercial and local genotypes, whose growth rates are not as fast as those of broilers. However, there was still a genotype effect on *IGF1R* gene expression between the commercial hybrid layer genotype and local genotype, reflecting their differences in growth rate and performance.

In addition, we evaluated *TLR4*, a transmembrane protein that is a member of the pattern recognition receptor family. It can also participate in the transmission of inflammatory signals, which cause the production of inflammatory substances. Our results indicated that Met in ovo feeding had no effect on *TLR4* expression in either genotype. However, the effect of the genotype was observed with HPC, which tended to increase *TLR4* expression relative to the TSL genotype. This indicates that HPC chicks are better protected against pathogens. This finding aligns with the trial reported on the hematological parameters of 28-day-old chicks from identical genotypes, which indicated that the HPC had high lymphocyte counts involved in both adaptive and innate immunity^5^.

Moreover, we analyzed the relationship between hepatic gene expression related to growth and immunity and hatching body weight as well as the organ weight of the chicks. Our analysis revealed that hepatic *IGF1, IGF1R, GHR and TLR4* were negatively correlated with the hatching body weight of the chicks. This indicates that heavy chicks had low expression of the analyzed genes, while lighter chicks had overexpressed growth genes and hence would have a high growth rate. This supports the result for the two genotypes, where HPC overexpressed growth-related genes more than TSL. Similar results were reported, with hepatic gene expression negatively correlated with body weight gain in other animal species^50^. In contrast, another study reported a positive correlation of hepatic *IGF1* and *IGF1R* expression with the body weight of chickens^51^.

## Conclusions

In conclusion, DL-methionine has advantages over L-methionine in promoting higher hatching weight, heart weight, and antioxidant capacity (FRAP) in the plasma of TSL and HPC genotypes. However, DL-methionine and L-methionine show similar effects on liver enzyme levels (AST, ALT), liver glutathione content, and plasma uric acid levels. This suggests that both Met sources can effectively support liver health and metabolism. The commercial genotype TSL appears to have improved the antioxidant defense system and enhanced hatching body weight, liver health, and function compared with the indigenous HPC genotype. This result demonstrates the difference in growth performance and metabolism between the two genotypes. The practical implications of these findings are significant for poultry management and nutrition and could potentially lead to enhanced breeding and production practices.

## Data Availability

All data are presented within the manuscript, and the raw data and associated analyses are available from the corresponding author upon reasonable request.

## Abbreviations

ACTB: Actin beta
AHW: Absolute heart weight
ALT: Alanine aminotransferase
ALW: Absolute liver heart
ANOVA: Analysis of variances
AST: Aspartate aminotransferase
Cys: Cysteine
DL-Met: DL-methionine
FRAP: Ferric reducing ability of the plasma
GHR: Growth hormone receptor
GSH: Glutathione
HBW: Hatching body weight
HPC: Hungarian partridge-colored chicken
IGF1: Insulin-like growth factor 1
IGF1R: Insulin-like growth factor 1 receptor
LD1: Linear discriminate function 1
LDA: Linear discriminator analysis
L-Met: L-methionine
Met: Methionine
Met-Cys: Methionine plus cysteine
MHA: Methionine hydroxyl analog
Cys: Cysteine
NRC: National Research Council
RHW: Relative heart weight
RLW: Relative liver weight
RMSE: Root square of mean of the standard error
ROS: Reactive oxygen species
RT‒PCR: Real-time polymerase chain reaction
TAC: Total antioxidant capacity
TLR4: Toll-like receptor 4
TSL: Tetra SL LL hybrid genotype
